# Credibility, Replicability, and Reproducibility in Simulation for Biomedicine and Clinical Applications in Neuroscience

**DOI:** 10.3389/fninf.2018.00018

**Published:** 2018-04-16

**Authors:** Lealem Mulugeta, Andrew Drach, Ahmet Erdemir, C. A. Hunt, Marc Horner, Joy P. Ku, Jerry G. Myers Jr., Rajanikanth Vadigepalli, William W. Lytton

**Affiliations:** ^1^InSilico Labs LLC, Houston, TX, United States; ^2^The Institute for Computational Engineering and Sciences, The University of Texas at Austin, Austin, TX, United States; ^3^Department of Biomedical Engineering and Computational Biomodeling (CoBi) Core, Lerner Research Institute, Cleveland Clinic, Cleveland, OH, United States; ^4^Department of Bioengineering and Therapeutic Sciences, University of California, San Francisco, San Francisco, CA, United States; ^5^ANSYS, Inc., Evanston, IL, United States; ^6^Department of Bioengineering, Stanford University, Stanford, CA, United States; ^7^NASA Glenn Research Center, Cleveland, OH, United States; ^8^Department of Pathology, Anatomy and Cell Biology, Daniel Baugh Institute for Functional Genomics and Computational Biology, Thomas Jefferson University, Philadelphia, PA, United States; ^9^Department of Neurology, SUNY Downstate Medical Center, The State University of New York, New York, NY, United States; ^10^Department of Physiology and Pharmacology, SUNY Downstate Medical Center, The State University of New York, New York, NY, United States; ^11^Department of Neurology, Kings County Hospital Center, New York, NY, United States

**Keywords:** computational neuroscience, verification and validation, model sharing, modeling and simulations, Simulation-Based Medical Education, multiscale modeling, personalized and precision medicine, mechanistic modeling

## Abstract

Modeling and simulation in computational neuroscience is currently a research enterprise to better understand neural systems. It is not yet directly applicable to the problems of patients with brain disease. To be used for clinical applications, there must not only be considerable progress in the field but also a concerted effort to use best practices in order to demonstrate model credibility to regulatory bodies, to clinics and hospitals, to doctors, and to patients. In doing this for neuroscience, we can learn lessons from long-standing practices in other areas of simulation (aircraft, computer chips), from software engineering, and from other biomedical disciplines. In this manuscript, we introduce some basic concepts that will be important in the development of credible clinical neuroscience models: reproducibility and replicability; verification and validation; model configuration; and procedures and processes for credible mechanistic multiscale modeling. We also discuss how garnering strong community involvement can promote model credibility. Finally, in addition to direct usage with patients, we note the potential for simulation usage in the area of Simulation-Based Medical Education, an area which to date has been primarily reliant on physical models (mannequins) and scenario-based simulations rather than on numerical simulations.

## Introduction

One hallmark of science is reproducibility. An experiment that cannot be reproduced by others may result from statistical aberration, artifact, or fraud. Such an experiment is not credible. Therefore, reproducibility is the first stage to ensure the credibility of an experiment. However, reproducibility alone is not sufficient. For example, an *in vitro* experiment is performed to advance or aid the understanding of *in vivo* conditions. However, the applicability of *in vitro* results to the living tissue may be limited due to the artifact of isolation: a single cell or a tissue slice extracted from its environment will not function in precisely the way it functioned *in situ*. In medicine, animal models of a disease or treatment are frequently used, but may not be credible due to the many differences between the human and the monkey, rat, or another animal that may limit the transfer of findings from one species to another.

In the computational world, the credibility of a simulation, model or theory depends strongly on the projected model use. This is particularly true when translating a model from research usage to clinical usage. In research, innovation and exploration are desirable. Computer models are used to introduce or explore new hypotheses, ideally providing a new paradigm for experimentation. In this setting, the most important models may in some cases be the less credible ones – these are the models that stretch understanding by challenging the common view of how a particular system works, in order to offer a paradigm shift. Here, *prima facie* credibility is in the eye of the beholder, who is more likely representing the views of the community – the dominant paradigm.

In the clinical realm, by contrast, establishing credibility is of paramount importance. For pharmaceuticals, credibility is currently established through evidence-based medicine (EBM), ideally through double-blind trials with large numbers of patients. The downside of this statistical approach is that it necessarily aggregates a large number of disparate patients to achieve statistical significance. In some cases, this has resulted in tragedy, as a subgroup with particular genetics has a fatal response to a drug that is beneficial in the overall group (e.g., rofecoxib, brand-name Vioxx). As EBM gives way to precision medicine, pharmaceutical credibility will be established in each subgroup, or even at an individual level, to enhance safety. However, to establish pharmaceutical reliability for personalized medicine (precision with a subgroup of *n* = 1), a lack of comparator precludes the use of data-mining by definition. Simulations based on patient genetics and various levels of epigenetics up through brain connectomics will then be the only way to predict the response of an individual patient to a particular treatment. Such patient simulation would provide a prediction of the response of that patient’s unique physiodynamics to a therapy. The credibility of such models will be essential.

In addition to pharmacotherapy, brain disease treatment also utilizes other therapeutic modalities, ranging from neurosurgery to the talk therapy of psychiatry and clinical psychology. While the latter will likely remain beyond the range of our modeling efforts, neurosurgery has already begun to benefit from modeling efforts to identify locations and pathways for epilepsy ablation surgery (Jirsa et al., 2017; Lytton, 2017; Proix et al., 2017). Another set of therapeutic approaches that are likely to benefit from modeling are the electrostimulation therapies that are finding increased use in both neurology and psychiatry, e.g., deep brain stimulation (DBS), transcranial magnetic stimulation (TMS), transcranial direct/alternating current stimulation (tDCS/tACS), and electroconvulsive therapy (ECT) (Kellner, 2012). Neurorehabilitation will also benefit from modeling to help identify procedures to encourage neuroplasticity at locations where change will have the greatest effect for relief of deficit.

In most respects, the issues of credibility, replicability, reproducibility for computational neuroscience simulation are comparable to those faced by researchers using simulation to study other organ systems. However, the complexity of the brain and several special features provide unique challenges. As with modeling of other organ systems, the relevant length scales range from molecular (angstrom) level up through tissue (centimeter) levels. Many experiments extend from the lowest to highest scales, for example evaluating performance on a memory task as a drug modifies ion channel activity at the molecular level. Compared to other organ systems, there is a particularly broad range of temporal scales of interest: 100 microseconds of sodium channel activation up to years of brain maturation and learning. In addition to physical scales of the central nervous system (CNS) itself, brain research includes further investigative levels of cognition, information, and behavior. An additional aspect of nervous system organization involves overlap between scales, which prevents encapsulation of one scale for inclusion in the higher scale. For example, spatiotemporal activity in apical dendrites of neocortical pyramidal cells (subcellular level) are co-extensive in both spatial and temporal scales with the scales of the local network (supracellular level).

In this paper, we will focus on the many issues of model credibility from a biomedical and clinical perspective. We will use *model* here forward to mean a mathematical model, primarily analyzed *in silico* via a *simulation*, which is the numerical instantiation of a mathematical model on a computer. We will identify explicitly in cases where we are discussing other types of models: verbal models, animal models, physical models, etc. Currently, there are still relatively few models of brain disease, and those remain in the research domain, rather than being practical clinical tools. Therefore, we are considering policy and practice for a future *clinical computational neuroscience*, based on the current uses of computational neuroscience in basic biomedical research, and on the clinical usage of simulations in other domains of medicine. In doing this, we will introduce some basic concepts that are important in the development of credible models: Reproducibility and Replicability; Verification and Validation.

## Reproducibility and Replicability

Replicability, here subsuming repeatability, is the ability to achieve a fully identical result (Plesser, 2017). For example, in the case of neuronal network simulation, a simulation has been fully replicated when one can show that spike times, as well as all state variables (voltages, calcium levels), are identical to those in the original simulation. Replicability is a design desideratum in engineering when one wants to ensure that a system being distributed runs identically to a prototype system (Drummond, 2009; Crook et al., 2013; McDougal et al., 2016a).

Reproducibility, in contrast, is the ability of a simulation to be copied by others to provide a simulation that provides the same *results* (Crook et al., 2013; McDougal et al., 2016a). This will then depend on what one considers to be a result, which will reflect the purposes of the simulation and will involve some measures taken on the output. Taking again the case of the neuronal network simulation, a result might involve some direct statistical measures of spiking (e.g., population rates), perhaps complemented by the summary statistics provided by field potential spectra, likely ignoring state-variable trajectories or their statistics.

Replicability and reproducibility are inversely related. A turnkey system, provided on dedicated hardware or a dedicated virtual machine, will run identically every time and therefore be fully replicable. However, such a system will not be reproducible by outsiders, and may in some cases have been encrypted to make it difficult to reverse engineer. Generally, the higher level the representation is, the more readily other groups understand a simulation and reproduce the results, but the less likely it is that they obtain an identical result – lower replicability. Representations using equations – algebra, linear algebra, calculus – are identical worldwide and therefore can provide the greatest degree of reproducibility by any group anywhere. However, reproducing a simulation from equations is a laborious process, more difficult than reproducing from algorithms, which is, in turn, more laborious than reproducing from a declarative (e.g., XML) representation. A dedicated package in the domain which provides a declarative description of a simulation will be more easily ported than general mathematical declarative description for \dynamical systems. Even at the level of reproduction from software code, differences in numerical algorithms used in computer implementations will lead to somewhat different results.

Different software representations also provide different levels of difficulty in the task of reproducing a simulation by porting to another language. The major innovation in this respects has been the development and adoption of object-oriented languages such as Java and Python. In particular, the use of object inheritance allows the definition of a type (e.g., a cell) with a subsequent definition of particular subtypes, where each type has parameter differences that are easily found and can be clearly documented by provenance.

Some difficulties with precise replicability of simulations are common to many different simulation systems. In particular, a model that uses pseudo-random numbers will not replicate precisely if a different randomizer is used, if seeds are not provided, or if randomizers are not handled properly when going from serial to parallel implementations. One difficulty peculiar to neural simulation is related to the strong non-linearities (and numerical stiffness) associated with action potential spike thresholding. Spiking networks are sensitive to round-off error; a single time-step change in spike time will propagate to produce changes in the rest of the network (London et al., 2010).

In general, specific simulation programs have enhanced reproducibility by providing purpose-built software to solve the particular problems of the computational neuroscience domain. Typically, these packages couple ordinary differential equation (ODE) solvers for simulating individual neurons with an event-driven queuing system to manage spike event transmission to other neurons in neuronal networks. These facilities are provided by neuronal network simulators such as BRIAN (Goodman and Brette, 2008), PyNN (Davison et al., 2008), and NEST (Plesser et al., 2015), which allow spiking neurons to be connected in networks of varying size. It should be noted that these simulators are very different from the artificial neural networks used in deep learning, which do not implement spiking neurons. Some other packages also add the ability to do more detailed cellular simulation for the individual neurons by adding the partial differential equations (PDE) needed to simulate the internal chemophysiology that complements the electrophysiology of spiking – NEURON (Carnevale and Hines, 2006) and MOOSE (Dudani et al., 2009) provide this additional functionality. Many computational neuroscience simulations in are still carried out using general-purpose mathematical software, e.g., Matlab (The Mathworks, Inc., Natick, MA, United States); or by more general computer languages such as FORTRAN, C, or C++, limiting their reproducibility, reusability, and extensibility. However, it should also be noted that the popularity of software also plays a role. A language or package that is widely used will increase the number of people that can easily contribute to developing or utilizing the simulations.

Since computational neuroscience simulations are often very large, extensibility to high-performance computing (HPC) resources is also desirable and will enhance reproducibility. Some current simulator tools offer a direct path to these larger machines. NetPyNE, a declarative language built on top of NEURON, provides a general description of a network that can be run directly either on a serial processor or, via MPI (message passing interface), on an HPC resource (Lytton et al., 2016). The Neuroscience Gateway Portal provides a shared, NSF-supported graphical resource that simplifies HPC for a variety of simulators, including NetPyNE, NEURON, NEST, BRIAN, and others, avoiding the need for the user to know MPI usage (Carnevale et al., 2014).

Hypothetically, journal articles permit reproducibility using equations given in the Methods section. Often, however, full details are not provided due to the enormous complexity of information associated with many different cell types, and complex network connectivity (McDougal et al., 2016a). Furthermore, parameters and equations may be given for one figure, but not for all figures shown. Journal articles may also have typographical or omission errors. And even when the document is complete and entirely without error, errors are likely to creep in when reproduction is attempted, and the model is typed or scanned back into a computer. For all of these reasons, an electronic version of a model is more accurate and more immediately usable than a paper copy. Some journals, and many individual editors or reviewers, require software sharing as part of the publication process. However, some authors resist this mandate, desiring to retain exclusive access to their intellectual property.

Databases of models and parts of models have become important and have encouraged reproducibility in computational neuroscience (Gleeson et al., 2017). Additional value is added by utilizing formal model definitions such as ModelML, CellML, NeuroML, VCML, SBML (Hucka et al., 2003; Zhang et al., 2007; Lloyd et al., 2008; Moraru et al., 2008; Gleeson et al., 2010; Cannon et al., 2014). Major databases are being provided by the Human Brain Project, the Allen Brain Institute, the International Neuroinformatics Coordinating Facility, and others. Other databases include the NeuroMorpho.Org database of neuronal morphologies, the ModelDB database of computational neuroscience models (see this issue) in a variety of simulation packages and languages, and the Open Source Brain database for collaborative research (Gleeson et al., 2012, 2015; Gleeson et al., unpublished). We discuss these resources further in the section discussing the “Role of the Community.”

A recent initiative to encourage reproducibility in science is the new *ReScience Journal* (rescience.github.io), which publishes papers that replicate prior computational studies. By hosting the journal on GitHub, new implementations are directly available alongside the paper, and alongside any ancillary materials identifying provenance or providing documentation. Recently, the classic Potjans-Diesmann cortical microcircuit model was ported from NEST to BRIAN, reproducing and confirming the primary results (Cordeiro et al., unpublished).

## Good Practices Contributing to Simulation Credibility

### Verification and Validation (V&V)

#### Verification

Verification and validation (V&V) help users demonstrate the credibility of a computational model within the context of its intended use. This is accomplished by assessing the quantitative and qualitative aspects of the model that influence model credibility. The process of establishing the model’s correctness and its capability to represent the real system is accomplished through the processes of verification, validation, uncertainty propagation, and sensitivity analysis. Of these, V&V represent the most well-known, and potentially confused, aspects of model assessment.

Computational models may be implemented using open-source or commercial (off-the-shelf) software, custom (in-house) code, or a combination of the two (modified off-the-shelf software). Verification assures that a computational model accurately represents the underlying mathematical equations and their solution on a specific code platform. Verification also emphasizes confirmation of parameter specification and the accurate translation of model data from model data sources into the model application. Full verification for all possible inputs is technically not possible due to the halting problem. Nonetheless, software implementation of model concepts should always include some level of code verification and, to the extent possible, follow best management practices and established quality-assurance processes. In the case of commonly used simulation packages, verification will be the responsibility of the platform developer and not of the user, but unanticipated usage can bring verification concerns back to the fore in particular cases.

Verification can be divided into two sequential steps: code verification and calculation verification. *Code verification*, initially performed by the software platform developer provides evidence that the numerical algorithms implemented in the code are faithful representations of the underlying physical or conceptual model of the phenomenon. Code verification should be repeated by the user in the case of novel usage of the simulator. Code verification establishes the reliability of the source code in representing the conceptual model, including relevant physics, for the problem. Ideally, benchmark problems with analytical solutions are employed to ensure that the computer code generates the correct solution at the specified order of accuracy. For example, a common reference in computational fluid dynamics (CFD) is laminar flow in a straight pipe with a circular cross-section, whose analytical solution is well-known (Bird et al., 1960; Stern et al., 2006). CFD modeling techniques are being used to provide insight into flow patterns and rupture-risk of cerebral artery aneurysms (Campo-Deaño et al., 2015; Chung and Cebral, 2015) and for interventional radiology planning (Babiker et al., 2013). Unfortunately, no analytic solutions are available for most computational neuroscience applications. Therefore, one is restricted to comparing results from one numerical approximation to that of another, without reference to any ground truth (Brette et al., 2007).

Next, *calculation verification* aims to estimate numerical and logical errors associated with the conceptual model implementation, i.e., the computational model representing the target application. Going back to the laminar pipe flow example, one would specify geometry, material properties, and loading conditions to match the problem at hand. This typically results in a problem that no longer has an analytical solution but must be solved numerically. Various aspects of the numerical representations, particularly discretization, are investigated and refined until the model is deemed to be accurate within a pre-specified tolerance. Upon completion of the calculation verification step, the developer has established (and should document) a bug-free implementation of the model concepts with reasonable parameter values.

One of aspect of verification that is often overlooked in the computational science community is the testing of model scripts and binary codes. Researchers tend to focus their attention on V&V in the context of overall program performance, but omit testing the functionality of individual software modules. Module verification can be implemented as a suite of tests which verify the functionality of individual functions and modules (unit tests) and their integration within the system (integration tests). It is common practice to automate the testing procedures using an automated testing framework to perform these tests after each version update.

#### Validation

Everyday vernacular often equates or confuses the terms “verification” and “validation.” As described in the previous section, *verification* seeks to ensure the quality of the implementation of the conceptual model. Meanwhile, *validation* seeks to assess how well the conceptual model and its implementation on a specified code platform represent the real-world system for a defined application. More rigorously stated, validation is an assessment of the degree to which the model and simulation is an accurate representation of the response of the real system within the intended context of use. In this case, validation is a comparative process that defines a qualitative or quantitative measure of how the model differs from an appropriate experimental or other data source – the *referent*, generally a series of experiments in our context. Validation also helps to ensure that the computational model has *sufficient* rigor for the context of use, although a more rigorous or more precise model is not necessarily more credible.

**FIGURE 1:**
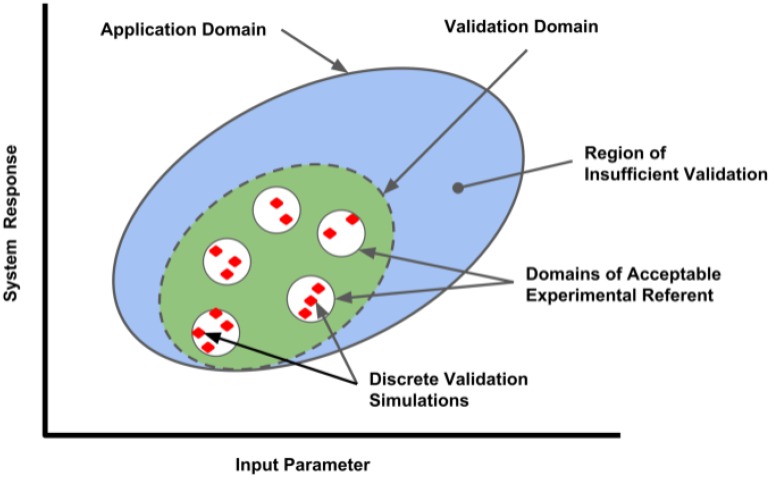
Validation domain of a computational model for a range of input parameters and outputs (system responses). Red diamonds represent the validation set points, where comparisons between the computational model and applicable referents were conducted by discrete simulations performed in each referent sub-domain. The range of parameters evaluated establishes the Validation Domain (green ellipse), which will define the extent of the Application Domain (large blue ellipse) where model performance has established credibility. Applications of the model outside of the Application Domain lack validation and have lesser credibility.

The definition of an appropriate referent is a critical aspect of model validation. Ideally, a validation referent consists of data obtained from a system with high similarity to the system being modeled in an environment similar to that of the target system. In clinical computational neuroscience, this is often difficult since data is obtained from a rodent (not high similarity to human), and sometimes from a slice (environment not similar to *in vivo* situation). The data used should be considered to be high quality by the model end-user community, and should represent data not used in model development. This separates *design data* from *testing data* (also called fit vs. benchmark data, or calibration vs. validation data). Model limitations due to inadequacies of the validation referent should be communicated. Practitioners should also keep in mind that a model validation, or the understanding of the variability of the model in predicting real world response, is only valid in the area of the referents used in the validation process, as illustrated by the Validation Domain (**Figure 1**). The Application Domain may be larger than the Validation Domain; it establishes the range of input and output parameters relevant to the context of use of the computational model. As the application of the model deviates from the situational context described by the referent, the influence of the model validation information will also change.

In most cases, the more quantitative the comparison, the stronger the case that a model and simulation is contextually valid. Organizations such as the American Society of Mechanical Engineers have developed standards to guide the model practitioner in performing successful model validation activities for specific application domains^1^. These comparisons range from qualitative, graphical comparative measures to quantitative comparative measures relying on statistical analysis of referent and model variances, the latter obtained over a wide range of input parameter variation (Oberkampf and Roy, 2010). Ideally, the end-user community and regulatory agencies will play a role in assessing the adequacy of the validation based on the context of an expected application and the influence the model has on critical decision-making.

### Aspects of Good Practice for Credibility

#### Software Aspects

The credibility of simulation results requires the reliability of simulation protocols and software tools. Good software practices include version control; clear, extensive documentation and testing; use of standard (thoroughly tested) software platforms; and use of standard algorithms for particular types of numerical solutions. It is also desirable to rely on existing industry standards and guidelines and to compare simulation results against competing implementations. To maintain the highest level of credibility, one would establish and follow these practices at every step of the development, verification, validation, and utilization of the simulation tools.

Version control is an approach for preserving model development and use histories, which can also be useful for tracing the provenance of model parameters and scope of applicability. There exists a large number of version control systems (VCS) which provide on-site, remote, or distributed solutions (e.g., Git, SVN, and Mercurial). In general terms, these systems provide tools for easy traceability of changes in individual files, attribution of modifications and updates to the responsible author, and versioning of specific snapshots of the complete system. Use of a VCS is recommended for both development (troubleshooting of bugs) as well as the day-to-day use of the modeling tools (monitoring of modeling progress).

Good documentation can be aided by the rigorous use of a detailed, dated electronic laboratory notebook (e-notebook). The e-notebook can provide automatic coordination with software versions and data output. E-notebooks are supported through various software packages, notably the Python Jupyter notebook and the Emacs org-mode notebook (Schulte and Davison, 2011; Stanisic et al., 2015; Kluyver et al., 2016). A major advantage over the traditional paper notebook is that the e-notebook can be directly integrated into simulation workflow, and will also provide direct pointers to simulation code, output, figures, data, parameter provenance, etc. This then allows later reviewers to identify all these links unambiguously. However, compared with a paper notebook, the e-notebook is at greater risk of falsification due to later rewriting. This risk can be reduced by including the e-notebook in the VCS, and can be eliminated by using blockchain technology (Furlanello et al., 2017). An e-notebook will also include informative records of model development and implemented assumptions; hypotheses and approaches to testing the hypotheses; model mark-up; detailed descriptions of the input and output formats; and simulation testing procedures. Going beyond the e-notebook, but also linked through it, the developer may add case studies, verification problems, and tutorials to ensure that other researchers and practitioners can learn to use the model.

It should be noted that even models developed following the aforementioned guidelines will have application bugs and usability issues. Thus, it is valuable to also cross-verify simulation results using alternative execution strategies and competing model implementations – e.g., run a simulation using BRIAN and NEST (Cordeiro et al., unpublished) – to reduce the chance of obtaining spurious simulation results. In addition to the inter-model verification, simulation process should be governed by generally applicable or discipline-specific standard operating procedures, guidelines, and regulations.

#### Developing Credible Mechanism-Oriented Multiscale Models: Procedure and Process

In science, an explanation can be inductive, proceeding from repeated observation. Ideally, however, explanation precedes prediction, permitting deductive reasoning (Hunt et al., 2018). Simulation of a mechanistic multiscale model provides an explicit way of connecting a putative explanatory mechanism to a phenomenon of interest.

Credibility and reproducibility can be enhanced by taking note of the many factors and workflows required to build a credible simulation to be used in a clinical application. One of these, often overlooked, is the role of exploratory (non-credible) simulations in building credible simulations. We would argue that most of the simulations that have been done in computational neuroscience are exploratory, and that we can now begin winnowing and consolidating these to create credible simulations for clinical application.

**FIGURE 2:**
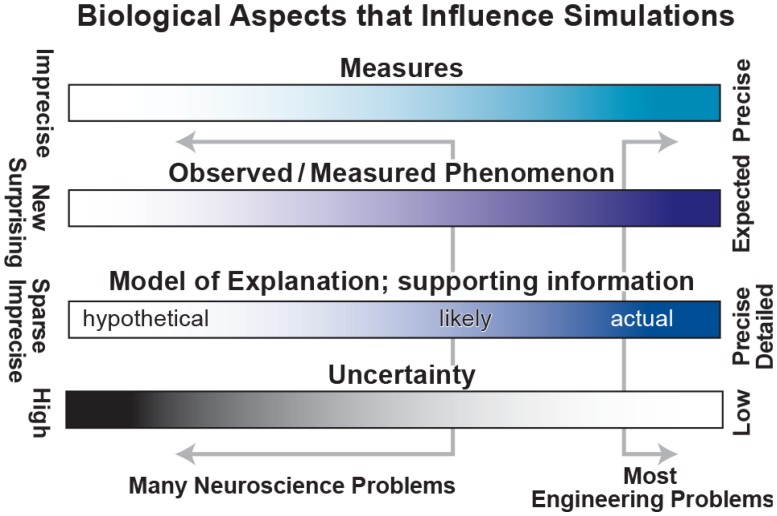
Spectra to characterize biological aspects of interest. Classical engineering problems lie at the right side of each range, working with precise measures, strong expectations, detailed information and low uncertainty. Unfortunately, most neuroscience problems lie far to the left with weak measures, unclear phenomenology, sparse information and high uncertainty.

Unfortunately, the problems in biology and particularly in neuroscience are characterized by (1) imprecise, limited measures – for example, EEG measures 6 cm of cortex at once (Nunez and Srinivasan, 2005); (2) complex observations whose relevance is sometimes unclear – there is no broad agreement on the relevance of particular bands of brain oscillations (Weiss and Mueller, 2012); (3) sparse, incommensurable and sometimes contradictory supporting information – for example, the difficulties of connecting microconnectomic (<1 mm; axon tracing, dual impalement, etc.) with macroconnectomic (1 cm; functional magnetic resonance imaging, fMRI) measures; and (4) high degree of uncertainty – parameter values obtained from brain slice work may differ from *in vivo* values. These limitations (left of ranges in **Figure 2**) contrast with the more solid information, concepts, observables and lower uncertainty associated with “classical” engineering of man-made devices such as computers, cars, and aircraft.

**FIGURE 3:**
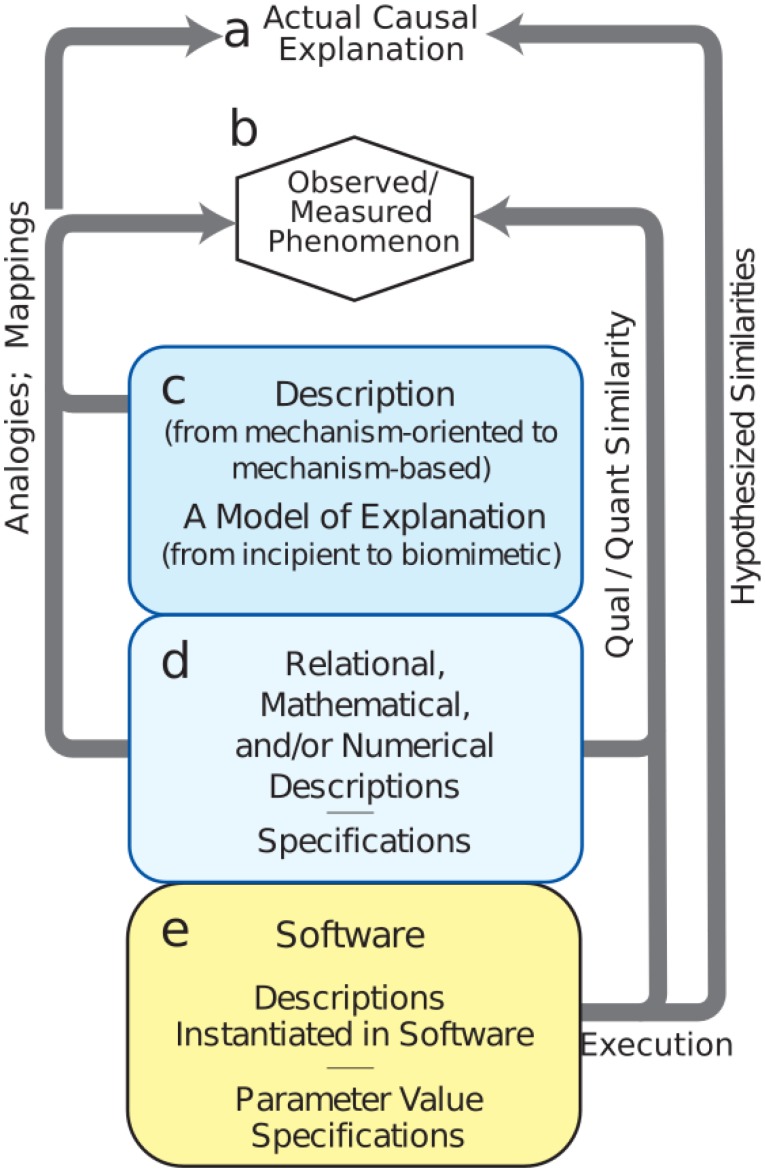
Mechanistic multiscale modeling process. **(a)** Causal explanation to be discovered. **(b)** Observables of phenomena to be explained. **(c)** Process (workflow) to identify and organize related information into descriptions; this process will involve simulation. **(d)** Meta-modeling workflows required for defining the extent of the final model. **(e)** Credible simulation which matches target phenomenon within some tolerance.

Model development is difficult, involving the need to consider and consolidate a large variety of factors from biology, engineering, mathematics, and design under the many constraints due to the limitations mentioned above. We have identified a set of procedures for the building of credible simulations, breaking out the many sub-workflows and processes involved (**Figure 3**). One can spend years building tools and running exploratory simulations that only lay the groundwork for future credible models. Nonetheless, it is important not to lose sight of the goal, which is to explain brain phenomenology at one or more levels of description: electrical rhythms, movement, behavior, cognition, etc.

The first task is to specify phenomena to be explained (**Figure 3b** – Observed Phenomenon). From this perspective, potentially relevant biological aspects are then organized together with relevant information and data into incipient explanations (**Figure 3c** – Descriptions and Explanations). In the computational neuroscience community, there are multiple perspectives regarding what information is to be considered relevant. For example, some argue that dendritic morphology and the details of ion channels are critical for understanding cortical networks (Amunts et al., 2017), while others consider that one need only consider simplified spiking cells (Diesmann et al., 1999; Potjans and Diesmann, 2014; Cain et al., 2016), and still others that it is best to work with high-level dynamical representations of populations (Shenoy et al., 2013) or mean-field theory (Robinson et al., 2002). Indeed all of these perspectives can be regarded as part of the explanatory modeling that will find its way into new concepts of (1) what is considered to be a relevant observation or measurement (**c** to **b** in **Figure 3**) and (2) what will be considered to be the form of an eventual causal explanation (**c** to **a**). Development of this incipient explanation will involve establishing mappings and drawing analogies between features of the explanation and particular measurements. These mappings and analogies may then be extended to provide working hypotheses and to actual preliminary biomimetic simulations for the eventual causal explanations.

A set of additional considerations (**Figure 3d** – Numerics and Specifications) provide the bridge from the exploratory activities (**Figure 3c** – Descriptions) to final credible models of (**Figure 3e** – Software). Although illustrated toward the bottom, these aspects of project formulation should be considered from the start as well. In computational neuroscience, potential use cases are still being developed and differ considerably across the four major clinical neuroscience specialties. Use cases, and data availability, will identify phenomena to be considered. For example, access to electroencephalography (EEG), but not electrocorticography, changes not only the type of software to be developed, but also the types of explanations to be sought. Use cases will also need to be organized based on expectations, separating near-term and long-term needs. New computational neuroscience users, use cases, and applications will arise in other specialties as they consider the innervation of other organs (Barth et al., 2017; Samineni et al., 2017; Vaseghi et al., 2017; Ross et al., 2018).

While identification of users and use cases remains relatively underdeveloped in computational neuroscience, the development of simulation tools is quite sophisticated. The need for multialgorithmic as well as multiphysics simulation has required that simulation platforms combine a variety of numerical and conceptual techniques: ODEs, PDEs, event-driven, graph theory, information theory, etc. Simulation techniques have been developed over more than a half-century, starting with the pioneering work of Hodgkin and Huxley (1952), Fitzhugh (1961), Rall (1962), and others. Today, we have a large variety of simulators with different strengths (Carnevale and Hines, 2006; Brette et al., 2007; Davison et al., 2008; Goodman and Brette, 2008; Bower and Beeman, 2012; Plesser et al., 2015; Tikidji-Hamburyan et al., 2017) that can be used individually or in combination, cf. MUSIC (Djurfeldt et al., 2010).

During the final stage of credible model development (**Figure 3e**), we demonstrate that the model provides the desired outputs to represent observations (**e** to **b**). A typical requirement is that simulation outputs agree with target phenomenon measurements within some tolerance. Since the simulation system will be multi-attribute and multiscale, it will at the very least begin providing mechanism-based, causal understanding across measures. To the extent that the model is truly biomimetic, direct mappings will exist to specific biological counterparts – voltages, spike times, field potential spectra, calcium levels, or others.

Although credible software appears to be the end of the road, actual practice will require that the resulting software system undergoes many rounds of verification, validation, refinement, and revision before even being released to users. From there, continued credibility requires continuing work on documentation, tutorials, courses, bug reports and bug fixes, requested front-end enhancements, and identified backend enhancements.

## Role of the Community

Community involvement is important to establish the credibility of the modeling and simulation process, and of the models themselves. The peer-review publication process serves as a traditional starting point for engaging the community. However, sharing of models, along with the data used to build and evaluate them, provides greater opportunities for the community to directly assess credibility. The role of sharing models and related resources has been acknowledged in the neurosciences community (McDougal et al., 2016a; Callahan et al., 2017). Related resources for sharing include documentation of the model (commonly provided in English) and implementation encoded in its original markup or code. With access to the model and its associated data and documentation, interested parties can then assess the reproducibility and replicability of the models and the simulation workflow first-hand. Further, reuse of the models and extensions of the modeling and simulation strategies for different applications can reveal previously unknown limitations. These various community contributions help build up the credibility of a model.

It is also important to note the value of cross-community fertilization to establish a common ground for credible modeling and simulation practices, to conform to and evolve standards that promote a unified understanding of model quality. Such standards enable individuals to easily exchange and reuse a given model on its own, or in combination with other models. Especially in multi-scale modeling, the ability to trust and build upon existing models can accelerate the development of these larger-scale efforts. Organizations such as the Committee on Credible Practice for Modeling and Simulation in Healthcare are leading efforts to establish such standards (Mulugeta and Erdemir, 2013).

Most community involvement in computational neuroscience has come through the establishment of databases and other resources that have encouraged submissions from the overall community of researchers. For example, the Scholarpedia resource established by Izhikevich and collaborators has hosted articles on computational neuroscience concepts and techniques that have been used to share concepts, modeling techniques, and information about particular modeling tools (Gewaltig and Diesmann, 2007; Wilson, 2008; Seidenstein et al., 2015). The Open Source Brain project provides a central location for collaborators working on modeling the nervous system of particular brain areas or of whole organisms, notably the OpenWorm project for modeling the full nematode nervous system (Szigeti et al., 2014). The Human Brain Project, an EU effort, has established a number of “Collaboratories,” web-accessible platforms to curate models and conduct simulations, to encourage community involvement in coordinated projects (Amunts et al., 2017). One of these projects aims to identify the parameters underlying individual synaptic events recorded in voltage clamp experiments (Lupascu et al., 2016). The Allen Brain Institute, another large modeling and data collection center, shares all results with the community, even before publication. Most of the major simulator projects encourage contributions from the community to provide either simulator extensions or additional analytic tools. For example, the SenseLab project hosts a SimToolDB alongside ModelDB for sharing general simulation code (Nadkarni et al., 2002). ModelDB itself is a widely used resource which specifically solicits model contributions and then provides a starting point for many new modeling projects that are extensions or ports of existing models (Peterson et al., 1996; McDougal et al., 2016b).

The above databases are used to provide completed models that are designed to be stand-alone but can also be used as components of larger models. By contrast, detailed neuron morphologies and ion channel models are generally only used as starting points for other models to build models at higher scales. Examples of these databases include the NeuroMorpho.Org database of neuronal morphologies and the Channelpedia database of voltage-sensitive ion channels (Ascoli et al., 2007; Ranjan et al., 2011).

The availability of these valuable resources is a testament to the successful engagement of the computational neuroscience community. A coming challenge will be to provide mechanisms for the discovery and selection of appropriate models for defined contexts among the existing hundreds of models. Here again, community involvement can play a critical role by providing the feedback and assessments of a model and its credibility to aid others in deciding whether to, or how to, re-use a model or part of a model.

## Use of Simulation in Medical Education

Simulation-Based Medical Education (SBME) is rapidly growing, with applications for training medical students, and residents (Jones et al., 2015; Yamada et al., 2017). However, the use of the word *simulation* in SBME differs from our usage above. SBME is referring to *simulated reality*: paper exercises based on protocols; mannequins; re-enactments with live actors for physical exams or major disasters; detailed computer-based virtual reality training, similar to video games. Currently, even the most advanced mannequins and computer-generated simulations have very limited capacity to produce a realistic focal neurological deficit or combination of signs and symptoms. Design and implementation of SBME tools is especially challenging in brain disease due to the complexity of the brain, and the variety of its responses to insult (Chitkara et al., 2013; Ermak et al., 2013; Fuerch et al., 2015; Micieli et al., 2015; Konakondla et al., 2017). This complexity suggests that back-end multiscale modeling would be particularly valuable in SBME development for brain disease.

Mechanistic multi-scale modeling has been used in both pre-clinical and clinical education to explain disease causality. For example, a demyelination simulation, relevant to multiple sclerosis and Guillain-Barre syndrome, is available that demonstrates both the effects of demyelination on action potential propagation and the consequent increase in temperature sensitivity (Moore and Stuart, 2011). Although this model has not been linked directly to a hands-on SBME activity, it could be linked to a training activity for Nerve Conduction Studies. Another promising area of recent research interest is in the area of neurorobotics, where robotic arms have been linked to realistic biomimetic simulations in the context of neuroprosthetics (Dura-Bernal et al., 2014; Falotico et al., 2017). In the clinical neurosciences, epilepsy is one of the most successfully modeled disorders in neurology and neurosurgery (Lytton, 2008). Jirsa et al. (2017) have pioneered models of individual patients prior to epilepsy surgery – see Section “Personalized Medicine Simulation for Epilepsy.”. These simulations could now be extended into training protocols for neurology and neurosurgery residents, offering an opportunity for melding educational simulation with computational neuroscience.

Current SBME efforts are focused on mannequins. A generic mannequin was used to train medical students to manage differential diagnoses and emergency procedures for status epilepticus and acute stroke (Ermak et al., 2013). The mannequin used was not capable of mimicking visible, or electrographic, signs of stroke or seizures. Instead, students were given chart data and simulation actors playing family member. Such simulations necessarily fall short on one aspect of effective implementation of SBME (Issenberg et al., 2005): the degree to which the simulation has a “real-life” feel.

In the future, life-like and highly immersive SBME will facilitate the learning of dangerous medical procedures, including emergencies and recovery from mistakes, in the way that is currently done with simulators used to train pilots, astronauts and flight controllers. Ideally, neurology and neurosurgery (and eventually psychiatry and physiatry) SBME systems will produce learned skills that are transferable to patient care. The predictive validity of the simulation could then be assessed by comparing performance measured under simulation conditions with corresponding measurements made on real patients (Konakondla et al., 2017). However, the skill level of a learner in the simulator should not be taken as a direct indication of real-life skill performance. In one example from flight training, a particular technique (full rudder), which served to “game” the simulator, resulted in a fatal accident due to tail separation when used in real life (Wrigley, 2013).

An example of SBME in neurosurgical training is the Neuro-Touch surgical training system developed by the National Research Council of Canada (Konakondla et al., 2017). The systems is built around a stereoscope with bimanual procedure tools that provide haptic feedback and a real-time computer-generated virtual tissue that responds to manipulation. As the surgeon is working through a surgical scenario, the simulator records metrics for detailed analysis to develop benchmarks for practitioners at different stages of training.

The Neurological Exam Rehearsal Virtual Environment (NERVE) virtual-patient SBME tool was developed to teach 1st- and 2nd-year medical students how to diagnose cranial nerve deficits (Reyes, 2016). Educational results were validated using questionnaires designed for virtual patient simulators (Huwendiek et al., 2014). An interesting future extension would be to provide an underlying simulator that would take account of the many complex neurological deficits found in patients due to the anatomical confluence of tracts in the brainstem. Such a simulator would be useful for neurology residents as well as for medical students.

To advance both the technologies and methodologies applied in SBME, the Society for Simulation in Healthcare (SSH) recently established the Healthcare Systems Modeling and Simulation Affinity Group. The Committee on Credible Practice of Modeling and Simulation in Healthcare (Mulugeta and Erdemir, 2013) has been collaborating with the SSH community by providing guidance on how to design and implement explicit multiscale computational models into traditional SBME systems. Additionally, the Congress of Neurological Surgeons has formed a Simulation Committee to create simulations for resident education (Konakondla et al., 2017). The US Food and Drug Administration (FDA) and the Association for the Study of Medical Education have also been working to publish standards and guidelines for regulatory submissions involving computational models and simulations for healthcare applications (Hariharan et al., 2017).

## Use of Modeling in Clinical Domains of Brain Disease

Simulations in the clinical neuroscience domain have largely focused on accounting for the neural activity patterns underlying brain disease. Testing the predictions arising from the simulation is dependent on technological advances in neuromodulation, pharmacology, electrical stimulation, optogenetic stimulation, etc. Neuropharmacological treatment is systemic, with effects wherever receptors are found, often peripherally as well as centrally. Although targeted treatment with an implanted cannula is possible, it is not widely used clinically. By contrast, electrical stimulation can be highly targeted with a local placement of electrodes. Development of closed-loop systems and devices for brain stimulation (Dura-Bernal et al., 2015), are currently being used and show promise in treating a wide range of neurological diseases and disorders including Parkinson, depression, and other disorders (Johansen-Berg et al., 2008; Shils et al., 2008; Choi et al., 2015). Non-targeted electrostimulation using transcranial electrodes is also being widely used but remains controversial, and lacks precise clinical indications (Lefaucheur et al., 2014; Esmaeilpour et al., 2017; Huang et al., 2017; Lafon et al., 2017; Santos et al., 2017). Consideration is also being given to future use of optogenetic stimulation therapies that would offer still greater precision compared to electrical stimulation – targeting not only a particular area but a particular cell type or set of cell types within that area (Vierling-Claassen et al., 2010; Kerr et al., 2014; Samineni et al., 2017). All such stimulation protocols require identification of suitable, if not necessarily optimal, ranges of parameters, e.g., strength, duration, frequency, waveform, location, iterations, schedule, reference activity, etc. It is not generally feasible to evaluate this large and high-dimensional stimulus parameter space empirically through experimentation. Hence, stimulation development would benefit from the extensive explorations possible using simulation of the response of micro- and macro-scale neural circuits in the brain, potentially in patient-specific fashion.

Successful application of models in the clinical domain will in future depend on the use of credible practices to develop, evaluate, document and disseminate models and simulations, using the principles outlined above. Nonetheless, clinical applications will also always need to be verified and validated by in a clinical before being utilized. At present, studies have been done in the absence of standards for computational neuroscience models. The current manuscript is designed in part to continue the discussion about the development of such standards. Meanwhile, clinically relevant studies have been performed with varying degrees of adherence to engineering best-practices. Here, we briefly discuss two such studies, noting adherence to such practices and where investigators may have fallen short.

### Simulation of Multi-Target Pharmacology

We first consider a mechanistic multiscale model of multi-target pharmacotherapy for disorders of cortical hyperexcitability (Neymotin et al., 2016). The study assessed hyperexcitability in an exploratory manner. They did not identify a single clinical context. Rather, they left the study open for exploration of cortical activation in both dystonia and seizures.

The multiscale model included molecular, cellular, and network scales, containing 1715 compartmental model neurons with multiple ion channels and intracellular molecular dynamics. Data used to develop the model was taken from a large number of sources including different species, different preparations (slice, cell culture, *in vivo, ex vivo*), different age animals, different states, different conditions. None of the data was taken from the clinical disorders in question due to limitations of human experimentation. As the model lacked a description of the motor output, the simulations could not be systematically evaluated in the context of dystonia. Beta activation in the cortex was used as a surrogate biomarker to evaluate whether simulations could account for activity patterns relevant to dystonia. However, as with many brain diseases, there is no established, clinically validated biomarker for dystonia. Additionally, the model lacked representations of spinal cord or limb, as well as many pharmacological parameters, particularly with respect to the role of neuromodulators (known unknown), brain states (less known unknown) and metabolic parameters.

The simulations were able to reproduce the target patterns of heightened cortical activity. The corresponding pathological parameter sets were identified by independent random variations in parameters. These simulations demonstrated degeneracy, meaning that there were many combinations of parameters that yielded the pathological syndrome. The primary result was that no individual parameter alteration could consistently distinguish between pathological and physiological dynamics. A support vector machine (SVM), a machine learning approach, separated the physiological from pathological patterns in different regions of high-dimensional space, suggesting multi-target routes from dystonic to physiological dynamics. This suggested the potential need for a multi-target drug-cocktail approach to intervening in dystonia.

Several aspects of best-practices were utilized in this study. Dissemination: The model was disseminated via publication and meeting presentations with the code made available via ModelDB resource (reference #189154). Documentation: Limited documentation was also made available at a level conforming to ModelDB requirements, consistent with practices accepted by the computational neuroscience community. Provenance: Due to the nature of the clinical domain, parameter provenance was partial; details of parameter sources were included in the paper. Replicability: Model replicability was tested by ModelDB curators, but was not tested directly by the manuscript reviewers. Reproducibility: There have not yet been any third-party studies reproducing the model. There are unexploited opportunities to compare the model with alternative implementations, for example by considering simpler modeling formalisms for single-neuron activity. The credibility of the simulations along with insights derived from the results would be enhanced by follow-on work that reproduces the simulations using similar or alternative implementations. Validation: The current lack of an adequate biomarker for dystonia limits the ability to validate this study in the future. Verification: The NEURON simulation platform was used in this study. It has been vetted both internally and in comparison to other simulators (Brette et al., 2007). Versioning: The Mercurial VCS was used to track parameter variations and match to corresponding simulations.

### Personalized Medicine Simulation for Epilepsy

As a contrast to above examination of the credibility practices as applicable to a mechanistic multiscale model, we considered these issues in the context an individualized phenomenological model of seizure propagation by Proix et al. (2017), implemented using The Virtual Brain platform (Sanz-Leon et al., 2013). The study was aimed at demonstrating that patient-specific virtual brain models derived based on information from diffusion MRI technique have sufficient predictive power to improve diagnosis and surgery outcome in cases of drug-resistant epilepsy. Data from individual patient tractography and EEG was utilized to parameterize each individual model separately. The diffusion MRI-based connectivity observed between the parcellated brain regions in each individual was used to create patient-specific connectivity matrices that related distinct autonomous oscillators (“Epileptors”) at each brain region. The resultant patient-specific virtual brain model was evaluated for its consistency in predicting seizure-like activity patterns in that patient.

Dissemination: The model and the results were disseminated as part of the published manuscript as well as through conference presentations and posters. Documentation: The platform is well documented. Internal documentation of individual models for use by neurosurgeons may be available but was not publically available, perhaps for reasons of patient confidentiality. Provenance: Provenance of connectivity data from individual patients was made clear. The Epileptor model is a phenomenological description of oscillatory patterns of activity in a bulk tissue (neural mass model); hence, there are no explicit parameters or variables that directly arise from specific molecular, cellular and metabolic pathways. Replicability: While the manuscript was peer-reviewed, it is not readily apparent if the model was tested directly in simulations by the manuscript reviewers. Reproducibility: Virtual Brain provides a particular dynamical formalism based on bulk activity. It would be interesting to see if alternative model formalisms that incorporate details at cellular scales would produce similar results. Validation: Alternative connectivity matrices and weightings were considered based on data from control subjects, shuffling the data, changing the weights while preserving the topology of the connectivity, and log-transformation. The authors demonstrated that prediction of seizure patterns was best when the patient-specific topology of the connectivity matrix was utilized. Verification: The study considered alternative models based on fast coupling, no time-scale separation, and a generic model that shows saddle-node bifurcation. Based on the simulations considering these alternative models, the authors concluded that weak coupling is necessary for the predictions on the recruited networks. The Virtual Brain platform has undergone considerable code testing over the years. Versioning: The platform is made available in a public repository using Git.

## Actionable Recommendations and Conclusions

The potential of modeling and simulation in clinical application and medical education are promising. However, this potential is mainly being tapped in the areas that are close to traditional engineering domains, such as CFD and stress analysis. For this reason areas of medicine that are related to blood flow, biomechanics and orthopedics have benefited most. By contrast, the brain has an idiosyncratic evolved set of mechanisms that are extremely difficult to reverse engineer and which draw on many areas of engineering, some of which have not been invented yet. Therefore, computational neuroscience remains primarily in the research domain, with only fragmented translations from computational neuroscience to clinical use or to medical education. As medical practice moves toward precision, and then personalized, healthcare, multiscale modeling will be necessary for simulating the individual patient’s response to disease and treatment. To move toward this goal, we must cultivate credible modeling and simulation practices taken from traditional areas of engineering.

### Model Configuration Management

Since many models will be built within the context of a simulation platform, we refer here to “Model configuration.” However, all of these points apply *a fortiori* to models being built from scratch in a general-purpose programming language.

(1) Use *version control*: Git or Mercurial (hg) are preferred. GitHub can be used to host projects (Dabbish et al., 2012). In shared projects, version control establishes who is responsible for which pieces of code.
(2) Use an *electronic notebook* (e-notebook) with clear documentation of every stage of model development (including mistakes).
(3) Include *provenance* of parameters in e-notebook and via version control – parameters may be changed due to new experimental data, and it is valuable to have a clear record of when and why the change was made and what the consequence was for the model.
(4) Perform testing of model components: for example, demonstrate that the cell-types show proper firing characteristics before incorporating them into networks.
(5) Later in the process, develop a test suite for further testing. Ideally, model testing should be performed at every version update. A test suite can be linked to standard testing frameworks to automate this testing. Commonly used frameworks include Travis CI (Continuous Integration), Circle CI, Jenkins, and AppVeyor.
(6) Whenever possible, use reliable model development platforms such as NEST, BRIAN, NEURON, MOOSE, NENGO, PyNN, etc. This will increase the likelihood of accurate simulation and will enhance sharing. Similarly, model components should be taken from reliable databases of morphologies, channels and other components.
(7) Later in the process, encourage other groups to compare simulation results on alternative platforms or with different implementations.

### Verify and Validate Models

(1) Simulation platform developers generally verify the adequacy of the numerical analysis. Some simulators offer alternative numerical solvers which can be tested to assess the qualitative similarity of results. For example, one problematic area is the handling of fixed and variable time steps for spike handling in networks (Lytton and Hines, 2005).
(2) Verify algorithms you develop. For example, when developing a neuronal network, make sure that the network design is correct before moving to actual simulation.
(3) Verify that the simulation is a reasonable implementation of the conceptual model, ideally by comparing a graphical output of basic phenomenology with target phenomena. It is tragically easy to move on to the analysis phase of the study without first looking at the raw output to make sure it is reasonable.
(4) Validate based on data from a real-world system. In some cases, it may be important to distinguish simulation results from the same model for different situations, e.g., *in vitro* slice vs. *in vivo* background activation.
(5) Test robustness of a model to parameter changes to ascertain whether a particular result is only seen with a particular set of parameter choices.
(6) Document and propagate V&V testing processes and findings to the community.

Although all of these steps are valuable, it is important to understand that higher model fidelity and V&V rigor do not automatically translate to higher credibility.

### Share Models and Data

(1) Submit models to shared databases such as ModelDB or via GitHub. Share widely – you can submit the same model to various databases, and submit components such as cells and ion channel definitions to component databases.
(2) Document thoroughly – the Methods section of a paper will provide an overall gloss but, typically, will not provide sufficient detail about software design and use. Comment code. Provide a README on how to launch the simulation.
(3) Disseminate – publish, of course, and present posters and talks to various audiences. In a multidisciplinary area such as computational neuroscience, the same model will say different things to different audiences – physiologists, anatomists, cognitive neuroscientists, neurologists, psychiatrists, other modelers.
(4) Join communities via organizations such as Society for Neuroscience (SFN), Computational and Systems Neuroscience (CoSyne), Organization for Computational Neuroscience (CNS).
(5) Obtain independent reviews of models. This is difficult and time-consuming but some grants are now providing funding explicitly to pay for consultants to review models (NIH, 2015).

### Define Context of Use and Simulation Requirements

We distinguished above between *exploratory models*, done by an individual researcher in order to provide ideas and new hypotheses, and *context models*, purpose-built models for external users in a given environment, for example, clinical or medical education use. In the latter case, it is essential to be clear about who the users are and which usage patterns (sets of use cases) are to be targeted and which ones are to be excluded or to be left as part of longer-term goals. However, even exploratory models can benefit from these considerations, envisioning yourself, your team, and perhaps an experimental collaborator as users.

(1) Identify the *users*. Even if you are the only user at the beginning of the project, you will be sharing the model later, so you may want to take account of other users who are not as familiar with your domain. For clinical use, a clinical assistant for epilepsy would be different for neurosurgeons vs. neurologists.
(2) Identify the *context of use*. For example, will this model primarily be used to study dynamics, or will it be extended into information theory or will it be expected to perform a memory function, etc.
(3) Identify *intended uses.* You may have one intended use for yourself as a modeler to generate new theoretical hypotheses and another for your experimental colleague. In the context of an educational application, an SBME for medical students will be very different than an application for residents or continuing medical education.
(4) Attempt to identify *usage patterns* – it is often the case that underprepared users who have not read the manual use a program in unintended, and sometimes dangerous, ways. Platforms and programs can produce warnings when it detects that the user is trying to use unsuitable combinations of parameters, etc.

### Translation of Computational Neuroscience Models for Clinical and Medical Education Use

In the preceding sections, we have given examples of particular clinical and educational/training scenarios that may be ripe for the introduction of simulation technology. Here we list both those already mentioned and others that have potential for future applications. This list is by no means complete.

(1) Education: Integration of modeling into mannequins and online virtual patients to reproduce neurological deficits in SBME. Initial versions of this would not require mechanistic multiscale modeling but could be done with phenomenological modeling. Future versions would incorporate mechanistic modeling to also incorporate the time-course of signs and symptoms (dynamics at multiple timescales).
(2) Training: Virtual reality simulators with haptic feedback for neurosurgery training.
(3) Personalized patient simulations to decide on surgery vs. interventional radiology (coiling) for aneurysms.
(4) Clinical decision making: Personalized patient simulations to decide on surgical approach for epilepsy surgery (Jirsa et al., 2017).
(5) Simulation for seizure prediction in Epilepsy Monitoring Unit (EMU).
(6) Personalized patient simulation to determine therapies for Parkinson disease to include combinations of surgical, electrical and pharmacological therapy (Grill and McIntyre, 2001; Hammond et al., 2007; Shils et al., 2008; Van Albada et al., 2009; Kerr et al., 2013; Holt and Netoff, 2017).
(7) Head, brain and neural modeling for understanding effects of different kinds of electrical stimulation including transcranial stimulation (Esmaeilpour et al., 2017; Huang et al., 2017; Lafon et al., 2017).
(8) Modeling vagal and peripheral nerve stimulation for treatment of systemic disorders (NIH SPARC program^2^).
(9) Psychiatry: identifying the varying roles of disorder in dopaminergic, glutamatergic, inhibitory and other deficits in schizophrenia to develop new multi-target therapies (Lisman et al., 2010).
(10) Neurorehabilitation (physiatry) Modeling the interface between neural and musculoskeletal models to treat spasticity or dystonia (van der Krogt et al., 2016).

## Author Contributions

LM and WL worked collaboratively to layout the contents of the manuscript, coordinate the team of co-authors, as well as edited the final draft of the manuscript. All of the co-authors drafted the specific section listed below beside their initials, as well as helped with the review and editing of the entire manuscript. LM: Abstract, Use of Simulation in Medical Education, and Actionable Recommendations and Conclusions, rewriting manuscript. AD: Software Aspects. AE and JK: Role of the Community. CH: Developing Credible Mechanism-Oriented Multiscale Models: Procedure and Process section. JM and MH: Verification and Validation (V&V). RV: Use of Modeling in Clinical Domains of Brain Disease. WL: Introduction, Reproducibility and Replicability, and Actionable Recommendations and Conclusions, rewriting manuscript.

## Conflict of Interest Statement

LM owns and operates InSilico Labs LLC and Medalist Fitness LLC. AE owns and operates Innodof, LLC. MH is employed by ANSYS, Inc. The other authors declare that the research was conducted in the absence of any commercial or financial relationships that could be construed as a potential conflict of interest.

## Footnotes

1 https://cstools.asme.org/csconnect/CommitteePages.cfm?Committee=100108782

2 https://commonfund.nih.gov/sparc
